# Stroboscopic visual training and the brain: a narrative review of neural mechanisms underlying perceptual-cognitive adaptation in interceptive sports

**DOI:** 10.3389/fnins.2026.1881779

**Published:** 2026-07-28

**Authors:** Xiushen Dong, Dongxu Gao, Yuqing Gao, Yong Guo, Tianci Hou, Congcong Weng, Jiayu Wang, Xinhong Zhou, Zixuan Hu

**Affiliations:** 1 School of Physical Education, Hunan Normal University, Changsha, China; 2 Division of Sports Science and Physical Education, Tsinghua University, Beijing, China; 3 School of Physical Education, Wuhan Sports University, Wuhan, China; 4 School of Competitive Sports, Beijing Sport University, Beijing, China; 5 Department of Physical Education and Military Training, Jiaxing University, Jiaxing, China; 6 School of Physical Education, Hubei University, Wuhan, China

**Keywords:** interceptive sports, neuroplasticity, predictive coding, stroboscopic visual training, visual evoked potentials

## Abstract

Stroboscopic visual training (SVT) is a perceptual-cognitive training paradigm that uses liquid-crystal eyewear to alternate between transparent and opaque states during sport-specific motor tasks, creating repeated cycles of vision and occlusion. By intermittently removing visual feedback, SVT compels the central nervous system to sustain internal predictions of moving targets rather than passively receiving real-time sensory input, placing concentrated and repeated demands on the predictive processing circuits that underpin performance in interceptive sports such as handball, volleyball, and soccer. Despite consistent behavioral evidence that SVT improves reaction speed, anticipatory skill, visuomotor performance, and sensorimotor balance control, the neural mechanisms driving these adaptations remain poorly characterized. This narrative review synthesizes evidence from SVT intervention studies, perceptual learning neuroscience, and sport neuroscience to propose a mechanistic account of how SVT may influence brain function. The review proposes that four neural systems may be engaged by SVT: the dorsal visual stream and MT/V5, which are repeatedly stressed by motion extrapolation demands during occlusion; the fronto-parietal attention network, which sustains predictive target representations across each occlusion cycle; the primary visual pathway, for which the only published SVT electrophysiology study demonstrated significantly reduced P100 latency following 6 weeks of stroboscopic training; and sensorimotor integration circuits, evidenced by SVT-induced changes in postural control and landing biomechanics. These findings are consistent with a proposed multi-level neuroplasticity model for SVT, though direct confirmation through concurrent EEG and fMRI designs remains an essential priority for future research.

## 1 Introduction

Athletes competing in interceptive sports face one of the most demanding information-processing environments in human movement science. Tracking a fast-moving ball, decoding opponent kinematics, and initiating a precise motor response must all occur within time windows that routinely fall below 300 milliseconds (Dingirdan Gultekinler et al., 2025; Huesmann et al., 2023). These temporal constraints mean that the perceptual-cognitive architecture of the brain is at least as important a determinant of performance as physical conditioning or technical skill (Brimmell et al., 2026; Panchuk et al., 2017). Experts consistently outperform novices not because their musculoskeletal systems are faster, but because their visual and cortical systems are better calibrated for generating accurate predictions before complete sensory information is available (Mackenzie et al., 2024; Muraskin et al., 2015).

Stroboscopic visual training (SVT) is a paradigm developed specifically to target these perceptual-cognitive demands. It uses liquid-crystal eyewear that alternates between transparent and opaque states at programmable frequencies, creating repeated cycles of vision and occlusion during otherwise normal sport-specific motor tasks (Ballester et al., 2017; Wilkins and Gray, 2015). SVT has been applied across a diverse range of interceptive sports, including handball (Zwierko T. et al., 2023), volleyball (Zwierko M. et al., 2023; Zwierko T. et al., 2024), soccer (Fortes et al., 2025; Zwierko M. et al., 2024), and table tennis (Strainchamps et al., 2023), and its application has extended into sensorimotor rehabilitation following musculoskeletal injuries (Grooms et al., 2018; Lee et al., 2022a; Mohess et al., 2024). This range of applications reflects a recognition that the visual occlusion mechanism at the heart of SVT is not sport-specific but targets a fundamental property of the central nervous system: its capacity to predict and extrapolate under conditions of incomplete sensory information (Harrison et al., 2024; Kim et al., 2017).

The neuroscientific rationale for SVT centers on what happens in the brain during each opaque interval. When visual feedback is transiently removed, the brain cannot passively read the current state of the environment but must instead generate a forward prediction of where a moving target will be when vision returns (Ballester et al., 2017; Cross et al., 2013). This forced reliance on internal predictive mechanisms is the mechanism that distinguishes SVT from other visual training approaches and aligns it directly with contemporary frameworks of predictive coding and action anticipation (Chen et al., 2020; Synofzik et al., 2013).

Despite this mechanistic appeal, the cortical and electrophysiological changes that SVT induces in the brain have received little direct investigation. Most published SVT research has documented behavioral outcomes, leaving the neural mechanisms that drive these gains unmapped (Dingirdan Gultekinler et al., 2025). This narrative review synthesizes available evidence from SVT experiments, perceptual learning neuroscience, and sport neuroscience to develop a mechanistic account of the visual, cortical, and perceptual-cognitive changes that SVT may produce in interceptive athletes.

A central caveat frames this review and should guide how its claims are read. Direct neurophysiological evidence obtained during or after SVT itself is currently limited to a small set of outcomes. On the electrophysiological side these are reduced P100 latency and motion-onset N2 changes, and on the biomechanical side they are altered postural control, ground reaction force, and muscle activation profiles. The higher-order cortical mechanisms that these findings are used to motivate, such as functional reorganization of motion-processing areas, recurrent laminar plasticity, sustained fronto-parietal prediction, and cerebellar forward-model refinement, have not been measured under stroboscopic conditions. They are instead inferred from adjacent research in visual perceptual learning, predictive coding, sport expertise, short-term sensory deprivation, and motor learning. To keep the status of each claim transparent, we distinguish three levels of evidence throughout the review. Findings described as empirically demonstrated in SVT were measured directly in a stroboscopic paradigm using physiological, electrophysiological, or biomechanical outcomes. Mechanisms described as theoretically plausible are well established in a closely related paradigm and are ones that the demands of SVT would be expected to engage, but that have not yet been tested under stroboscopic conditions. Interpretations described as speculative are offered on the basis of broader analogy, and are intended to generate hypotheses rather than to describe established effects. We signal these levels with calibrated language: “has been shown” or “demonstrated” for the first level, “may,” “is hypothesized to,” or “is consistent with” for the second, and “might” or “we speculate” for the third. Table 1 then assigns each proposed plasticity mechanism to its evidence tier and states whether the supporting data come from an SVT study or from the wider literature.

**Table 1:** Neural mechanisms proposed to underlie stroboscopic visual training adaptation.

Proposed neural mechanism	Primary brain region	Supporting evidence	SVT condition that engages it	Evidence tier
Accelerated early visual pathway transmission	Retina → V1 (primary visual cortex)	Zwierko T. et al. (2023)	6-wk sport-specific SVT; extra-foveal dominant eye condition	Demonstrated (direct SVT evidence): P100 implicit time reduced post-SVT (VEP)
N2 motion onset VEP acceleration (visual perception speed)	Occipital / extrastriate (N2, N2-r components)	Hülsdünker et al. (2021)	10-wk badminton SVT; visual motion stimulus response task	Demonstrated (direct SVT evidence): N2/N2-r latency changes explained >60% of VMRT variance post-SVT (EEG)
Feedforward plasticity in early visual cortex (V1 → V3A)	V1, V3A (extrastriate dorsal stream)	Song et al. (2024), Jia et al. (2020)	Repeated motion encoding across each SVT occlusion cycle	Plausible (inferred from perceptual learning literature)
Functional reorganisation of motion-processing areas	MT/V5, V3A	Chen et al. (2016), Ni et al. (2023)	Progressive occlusion difficulty increasing MT reconstruction demand	Plausible (inferred from perceptual learning literature)
Homeostatic upregulation of visual cortex responsiveness	V1, striate cortex	Lyu et al. (2020)	Brief repeated occlusion cycles per SVT session	Plausible (inferred from monocular deprivation literature)
Fronto-parietal prediction maintenance across occlusion gaps	DLPFC, posterior parietal cortex	Yan et al. (2016), Mishra et al. (2015)	Sustained target tracking during each SVT occlusion interval	Plausible (inferred from perceptual training literature)
Cerebellar forward model refinement for interceptive timing	Anterior cerebellum	Chen et al. (2022), Cross et al. (2013)	Interceptive timing predictions over repeated SVT occlusion cycles	Plausible (inferred from sport neuroscience literature)
Premotor time shortening (motor planning acceleration)	Premotor cortex (BA6)	Hülsdünker et al. (2021)	10-wk SVT in badminton; EMG onset during VMRT task	Demonstrated (direct SVT evidence): premotor not motor time shortened post-SVT (EMG)
Sensory reweighting (increased proprioceptive gain)	Sensorimotor cortex, vestibular nuclei	Kim et al. (2017), Lee et al. (2022b)	Balance and landing tasks performed under SVT conditions	Demonstrated (direct SVT evidence): altered visual reliance and postural control (force plate)
Sensorimotor feedforward motor command modification	Motor cortex, spinal motor pathways	Harrison et al. (2024), Grooms et al. (2018)	Landing/jumping tasks with stroboscopic visual disruption	Demonstrated (direct SVT evidence): GRF, EMG, and knee kinematics altered under SVT (biomechanics)

DLPFC, dorsolateral prefrontal cortex; MT/V5, middle temporal area; VEP, visual evoked potential; EMG, electromyography; VMRT, visuomotor reaction time; GRF, ground reaction force. Each mechanism is assigned to one of the three evidence tiers defined in the Introduction. “Demonstrated” means the mechanism was measured directly within an SVT study using physiological, electrophysiological, or biomechanical methods. “Plausible” means the mechanism is inferred from the broader perceptual learning, sensory neuroscience, or sport neuroscience literature and has not yet been confirmed under stroboscopic conditions. Mechanisms that rest on broader analogy alone, such as pulvinar-mediated attentional gating and sleep-dependent consolidation, are discussed in the text and identified there as speculative.

As this is a narrative rather than a systematic review, we aimed for a conceptually representative rather than exhaustive body of evidence and did not follow PRISMA procedures. We searched PubMed, Web of Science, Scopus, and Google Scholar for records published up to May 2026, combining the terms “stroboscopic,” “strobe,” “intermittent visual occlusion,” and “stroboscopic vision” with “training,” “sport,” “perceptual-cognitive,” “reaction,” “anticipation,” “agility,” and “postural control.” This primary search identified the empirical SVT intervention and acute-exposure studies that form the evidential core of the review and are summarized in Table 2. Because direct neurophysiological investigation of SVT is scarce, we then ran targeted secondary searches for the adjacent literature needed to interpret these findings, spanning visual perceptual learning, predictive coding, short-term sensory deprivation, sport expertise neuroscience, and motor learning. Supporting references were selected by author consensus on the basis of conceptual relevance to a proposed SVT mechanism, methodological quality, and the use of neurophysiological or neuroimaging measurement (EEG, ERP, MEG, fMRI, or biomechanics), and were supplemented by backward and forward citation tracking of key papers. Inclusion was restricted to peer-reviewed, English-language sources. Throughout the review, studies from these adjacent fields are used to generate and constrain hypotheses about SVT mechanisms rather than as direct evidence of them.

**Table 2:** Summary of published stroboscopic visual training intervention studies.

Study	Sport/context	*n*	SVT protocol	Strobe condition	Key outcome	Neural/biomechanical measure
*Zwierko T. et al. (2023)	Handball	22	6 wk	Sport-specific drills	Reduced P100 implicit time (extra-foveal, dominant eye; foveal dominant eye binocular)	VEP (P100)
*Hülsdünker et al. (2021)	Badminton	32	10 wk	Sport-specific drills	N2 / N2-r latency changes explained >60% of VMRT improvement; accelerated visual perception post-SVT	EEG (N2, N2-r, Vlc)
Zwierko M. et al. (2023)	Volleyball	50	6 wk	Sport-specific tasks	Complex reaction speed (d = 0.87); simple motor time (d = 0.42); reactive agility improved	None
Zwierko T. et al. (2023)	Volleyball	50	6 wk	Agility-based drills	Visuomotor reaction speed improved (*d* = 0.87); saccade acceleration correlated with gains (*r* = −0.28)	None
*Hülsdünker et al. (2021)	Badminton	32	10 wk. + 6 wk. retention	Sport-specific drills	VMRT reduced (pre 251 ms vs. post 238 ms; d = 0.63); premotor time shortened; retained at 6 wk	EMG onset
Fortes et al. (2025)	Soccer	28	8 wk	Sport-specific tasks	Anticipation skill improved vs. control (*p* < 0.05); no group effect for decision-making or MOT	None
Zwierko T. et al. (2024)	Soccer	22	Acute (warm-up)	Ball dribbling tasks	Reactive agility improved with ball (ES = 0.57); no effect without ball; effect retained under fatigue	None
Wilkins and Gray (2015)	Catching	30	6 wk. (9 sessions)	Ball-catching drills	UFOV and motion-depth sensitivity changes correlated with catching performance gains	None
Bennett et al. (2018)	Lab (MOT/MOA)	Exp	Single session	Vapor Strobe vs. PLATO occlusion	Vapor Strobe maintained MOT performance; facilitated MOA skill acquisition; PLATO impaired performance	None
Strainchamps et al. (2023)	Table tennis	28	Acute (warm-up)	Sport-specific warm-up	Warm-up improved RT; stroboscopic condition added no benefit over normal warm-up	None
Ballester et al. (2017)	Lab (CA task)	20	Single session	Coincidence-anticipation task	SVT prevented vigilance decrement across trial blocks; performance maintained vs. normal vision	None
*Kroll et al. (2023)	Volleyball	13	Single session	Depth jump (4 Hz; 1.75 Hz)	RSI and jump height reduced at 1.75 Hz; GRF development rate increased; SVT modifies plyometric intensity	GRF
*Harrison et al. (2024)	Lab (landing)	20	Single session	Depth jump (3 Hz strobe)	Peak GRF + 6.4%; tibialis anterior EMG − 14%; vastus lateralis EMG − 12%; altered feedforward motor commands	EMG / GRF
*Grooms et al. (2018)	Rehabilitation	30	Single session	Drop vertical jump	ACLR group showed altered sagittal-plane knee flexion compared to controls under stroboscopic disruption	Kinematics

SVT, stroboscopic visual training; VMRT, visuomotor reaction time; MOT, multiple object tracking; MOA, multiple object avoidance; UFOV, useful field of view; CAI, chronic ankle instability; ACLR, anterior cruciate ligament reconstruction; GRF, ground reaction force; EMG, electromyography; ES, effect size; d, Cohen’s d; RT, reaction time; ML, medial-lateral; EO, eyes open; EC, eyes closed; wk, weeks; Exp, experiment (within-subjects). Studies are ordered from highest to lowest neurophysiological evidence. *indicate that neurophysiological or biomechanical measures were obtained.

For clarity, we use several related terms with distinct meanings throughout. Neuroplasticity is the umbrella term for any experience-dependent change in neural structure or function; neural adaptation and cortical reorganization refer to specific forms of that change, the former denoting altered responsiveness of neurons or circuits and the latter denoting a shift in which regions or layers carry out a function; neural efficiency denotes equivalent or better performance achieved with reduced or more focused cortical activation; and sensory reweighting denotes a shift in the relative reliance the nervous system places on visual, proprioceptive, and vestibular inputs.

## 2 The predictive coding account of stroboscopic visual training

Predictive coding offers the most direct theoretical account of why intermittent visual occlusion should train the brain, and it is invoked throughout this review as the organizing framework. In this view, the cortex is not a passive receiver of sensory input but a hierarchical inference system that continuously predicts its own incoming signals. Higher cortical levels generate top-down predictions of the activity expected at lower levels, and lower levels return only the difference between the predicted and the actual input, a residual signal known as prediction error. Perception, on this account, is the ongoing process of minimizing prediction error by revising internal predictions until they match sensory evidence (Rao and Ballard, 1999; Friston, 2010; Clark, 2013). Stroboscopic visual training maps onto this architecture in a specific way. During the transparent phase, the moving target continuously supplies bottom-up input, prediction errors are available on every frame, and the estimate of target position is corrected almost immediately. During the opaque phase, this bottom-up input is removed and the brain has no sensory evidence against which to check its estimate, so it must instead run its internal generative model forward, extrapolating the target trajectory from the last available information and from learned regularities of how targets move. When vision returns at the start of the next transparent phase, the discrepancy between the extrapolated position and the true position generates a large, behaviorally relevant prediction error that the system can use to correct its estimate and, over repeated cycles, to improve the model that produced it.

Two features of the framework are particularly relevant to interceptive sport and to the neural systems reviewed below. The first is that prediction operates across a cortical hierarchy rather than at a single stage. Low-level areas such as V1 and MT/V5 predict local visual features and motion, while higher-level parietal and frontal regions predict the extended trajectory of the target, the likely intention of the opponent, and the appropriate motor response, with predictions passing downward and prediction errors passing upward between levels (Bastos et al., 2012). This hierarchical organization is why a single occlusion manipulation can plausibly engage early visual, dorsal-stream, fronto-parietal, and cerebellar circuits at the same time, as the following sections describe. The second feature is precision weighting. The brain does not treat all prediction errors equally but weights each one by its estimated reliability, or precision, and this weighting is widely regarded as a candidate neural implementation of attention (Feldman and Friston, 2010; Summerfield and de Lange, 2014). Stroboscopic training can therefore be understood as a direct manipulation of sensory precision. When the eyewear becomes opaque, the precision of visual input falls to zero, so the system is compelled to down-weight the absent visual evidence and to up-weight both its internal predictions and the remaining proprioceptive and vestibular signals. This provides a principled explanation for the sensory reweighting observed during stroboscopic balance and postural tasks, which is otherwise described only phenomenologically.

The framework also specifies how repeated exposure could produce lasting adaptation, which is the central claim of any training intervention. Prediction errors are not used only to correct the current percept; when they recur systematically, they drive updating of the generative model itself through experience-dependent synaptic plasticity, so that future predictions become more accurate and are generated with less residual error (Friston, 2010). Each stroboscopic cycle delivers exactly this kind of teaching signal, because the reappearance of the target at the end of every occlusion interval provides an unambiguous comparison between what the brain predicted and what actually occurred. Across the dozens of cycles in a single session and the many sessions of a multi-week program, this repeated error-driven updating would be expected to sharpen the forward models used for motion extrapolation and interceptive timing, and to make their neural signaling faster and more efficient. This offers a mechanistic interpretation of the electrophysiological changes reviewed in the following sections, in which early error signaling becomes faster, indexed by reduced P100 and N2 latency, and discrimination becomes more efficient, and it links these markers to the perceptual learning plasticity that has been demonstrated in early and extrastriate visual cortex under comparable training demands.

Predictive coding is not the only framework capable of explaining the effects of stroboscopic training, and a balanced account requires acknowledging alternatives. The most closely related is the internal forward model tradition from sensorimotor control, in which the cerebellum and associated circuits use an efference copy of the motor command to predict the sensory consequences of action (Wolpert et al., 1995; Shadmehr et al., 2010). This account overlaps substantially with predictive coding but places its emphasis on sensorimotor prediction rather than perceptual inference, and it is best regarded as complementary, since the cerebellar contributions discussed later are most naturally described in forward-model terms. A second alternative attributes the benefits of stroboscopic training not to updating of a generative model but to trained allocation of visual attention, such that occlusion simply teaches athletes to distribute attention more effectively under degraded input. A third and more conservative possibility is that some reported gains reflect non-specific factors, including heightened arousal, effort, motivation, or the general novelty and difficulty of training under unusual visual conditions, rather than any occlusion-specific mechanism. These accounts are not mutually exclusive, and the current stroboscopic training literature does not contain the concurrent neural and behavioral data needed to decide between them. We adopt predictive coding as the primary organizing framework because it connects the specific occlusion manipulation to the full pattern of observed effects more completely and more parsimoniously than the alternatives, while emphasizing that it remains a framework for generating and constraining hypotheses about stroboscopic training rather than a mechanism that has been directly demonstrated within it.

## 3 Visual processing and the brain: neural pathways targeted by stroboscopic visual training

Understanding the neural mechanisms of SVT requires identifying which cortical pathways it most directly engages. The visual system is organized into two largely segregated processing streams originating in primary visual cortex (V1). The dorsal stream projects toward posterior parietal cortex and is specialized for spatial localization and visually guided motor action. The ventral stream extends toward inferotemporal cortex and supports object recognition and fine-grained form processing (Chen et al., 2016; Song et al., 2024). SVT primarily engages the dorsal stream because the core demand during occlusion intervals is to maintain and update a spatial representation of a moving target, not to identify it (Bridge et al., 2014). This spatial tracking demand persists through the opaque phase and must be resolved before vision returns, placing a sustained and repeated load specifically on the motion-processing and action-guiding circuitry of the dorsal pathway.

The middle temporal area (MT/V5) is the primary cortical site for encoding visual motion and contains neurons tuned to the direction and speed of moving stimuli (Bridge et al., 2014; Jeschke et al., 2023). During the opaque phase of SVT, MT/V5 loses its primary sensory drive and is hypothesized to reconstruct a motion signal from residual activation and top-down predictions, which would generate a forward extrapolation of the target trajectory (Ballester et al., 2017). The contribution of MT to motion perception is not fixed but depends on training history: with repeated practice on a motion task, readout of motion information can shift from MT toward earlier visual areas, or from MT toward V3A, depending on the specific demands of the trained task (Chen et al., 2016; Ni et al., 2023). SVT, by repeatedly interrupting and then reinstating visual motion input across dozens of cycles per session, may provide the kind of experience-dependent signal thought to drive this form of cortical reorganization. It should be emphasized that this form of reorganization has been demonstrated in perceptual learning paradigms rather than in SVT itself, and its occurrence under stroboscopic conditions is therefore a prediction of the present framework rather than an established finding. Research using audio-visual multisensory training has further confirmed that targeting the colliculo-dorsal MT pathway through non-standard sensory conditions can specifically enhance the N1 ERP component during motion discrimination, demonstrating that non-standard stimulation modes can directly modify activity in motion-processing extrastriata areas (Grasso et al., 2016).

SVT also engages sensory reweighting mechanisms that extend beyond the purely visual cortical hierarchy. When visual input is transiently removed during SVT, the brain compensates by increasing reliance on proprioceptive and vestibular signals to guide ongoing movement (Kim et al., 2017). Research using stroboscopic glasses during postural control tasks has demonstrated that stroboscopic vision alters visual reliance in a graded fashion, inducing an intermediate condition between full vision and complete eye closure that is particularly effective when somatosensory inputs are simultaneously disturbed by an unstable surface (Harrison et al., 2024; Lee et al., 2022a). This sensory reweighting during SVT may itself be a source of neural adaptation, progressively improving the brain’s capacity to integrate motor predictions with non-visual sensory information when visual feedback is unreliable.

**Figure 1:**
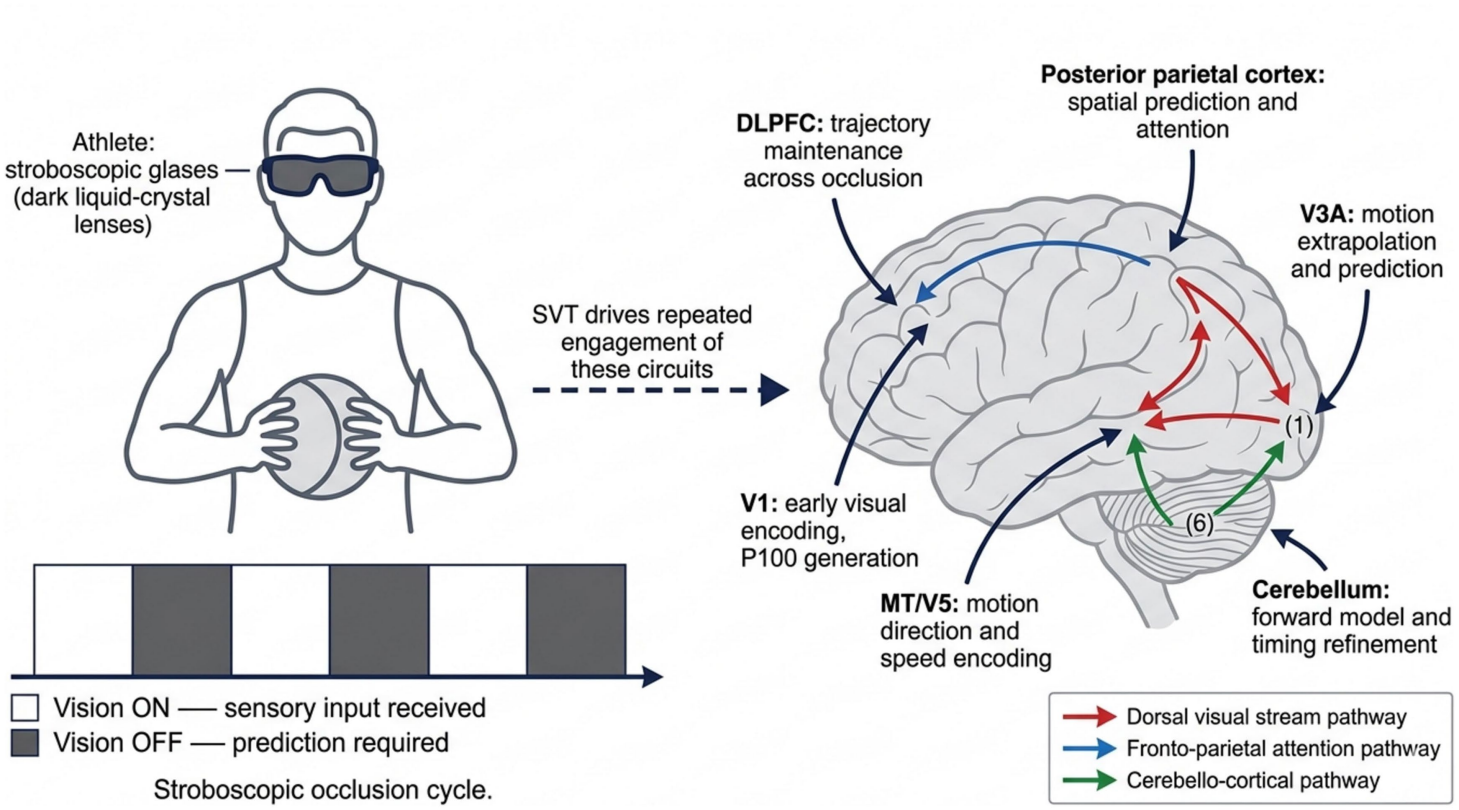
Conceptual framework of stroboscopic visual training and its neural targets. This figure presents a proposed, hypothesis-generating model of how a single stroboscopic cycle is thought to engage the dorsal visual stream and MT/V5, fronto-parietal attention networks, and cerebellar forward-model circuits during the transition from vision to occlusion and back. It is intended to organize the mechanisms discussed in this review and to guide future research, not to depict an experimentally established circuit. With the exception of the P100, N2, and biomechanical outcomes summarized in Table 1, the pathways shown have not been measured directly under stroboscopic conditions and are inferred from adjacent literatures. Arrows denote proposed functional relationships rather than confirmed anatomical or causal connections.

At the level of attention and executive control, the fronto-parietal network plays a critical role in maintaining object representations across the temporal gaps that SVT creates. Transcranial magnetic stimulation studies have dissociated the roles of the dorsolateral prefrontal cortex (DLPFC) and posterior parietal cortex (PPC): the DLPFC mediates strategic top-down target selection under high cognitive load, while the PPC handles reflexive attentional capture by sudden motion signals (Yan et al., 2016). During SVT, both mechanisms are simultaneously engaged, as the PPC must rapidly re-engage with the target when vision returns and the DLPFC must maintain the trajectory prediction throughout each occlusion interval (Mishra et al., 2015). The pulvinar nucleus of the thalamus coordinates and weights excitatory communication between frontal and parietal attention regions (Guedj and Vuilleumier, 2023), a role that would be relevant under SVT conditions where top-down predictions and bottom-up motion signals must be rapidly integrated. We note, however, that no SVT study has examined pulvinar activity, and its involvement is therefore proposed here on theoretical grounds alone and remains speculative. Sport expertise research using fMRI has shown that elite athletes performing multiple object tracking tasks exhibit reduced frontal and parietal activation for equivalent perceptual performance, consistent with neural efficiency gained through years of domain-relevant perceptual experience (Qiu et al., 2019). SVT may accelerate the development of this efficiency by repeatedly stressing the fronto-parietal prediction system. Figure 1 presents a conceptual framework, offered as a working hypothesis rather than an established model, of how intermittent visual occlusion is proposed to engage the dorsal visual stream, fronto-parietal attention networks, and cerebellar forward-model circuits during a single stroboscopic cycle.

## 4 Electrophysiological evidence: EEG and ERP markers relevant to stroboscopic visual training

Before reviewing this evidence, we state its balance plainly. Direct electrophysiological data recorded under stroboscopic conditions currently come from only two studies: a controlled visual evoked potential study in handball players (Zwierko M. et al., 2023) and a study in badminton players that recorded motion-onset N2 and electromyographic markers (Hülsdünker et al., 2021). The remainder of this section draws on perceptual training and sport neuroscience research in which the relevant components were recorded outside any stroboscopic paradigm. These related findings are used to predict which electrophysiological changes SVT might produce and to interpret the two direct results above; they are not themselves evidence that these components have been observed during or after SVT. The most direct evidence linking SVT to cortical change comes from the controlled VEP study. Zwierko (Zwierko M. et al., 2024) assigned 22 elite handball players to an SVT group or a matched control group performing identical sport-specific tasks under normal vision over 6 weeks. VEP recordings showed significant reductions in P100 implicit time for the SVT group in extra-foveal vision conditions for the dominant eye and for binocular viewing, with no comparable change in controls. The P100 component, a positive deflection peaking approximately 100 ms after a visual stimulus, reflects the speed of signal transmission from retina to occipital cortex through the primary visual pathway. Faster P100 latencies indicate more efficient early visual processing, meaning that SVT appears to accelerate the initial cortical encoding of visual information (Zwierko T. et al., 2023). The specificity of improvements to extra-foveal vision is particularly notable for interceptive athletes, who must frequently track objects through peripheral regions of the visual field rather than directly at the fovea.

Although no other published SVT study has directly recorded ERP components during or after training, related electrophysiological research from perceptual training and sport neuroscience provides a framework for interpreting what additional SVT-induced changes might look like. The N2 component, a negative deflection peaking between 200 and 350 ms post-stimulus, is broadly associated with stimulus discrimination, conflict monitoring, and detection of novelty in the visual stream. Covey et al., 2019 demonstrated that perceptual discrimination training produces earlier N2 latencies and modified amplitudes in healthy adults, consistent with more efficient conflict resolution. Mishra et al. (2015) showed that N1 and N2 amplitudes were significantly enhanced after training on rapidly presented perceptual tasks, with N2 enhancement specifically linked to improved discrimination of stimuli presented at challenging temporal rates. Because SVT repeatedly presents visual targets at intermittent intervals that force rapid post-occlusion target reacquisition, the stroboscopic cycle may function as a repetitive training stimulus for exactly the neural discrimination mechanisms indexed by N2. Takahashi et al. (2025) found that N2 amplitude was significantly reduced following acquisition of a fine visuomotor skill, interpreted as evidence of more efficient stimulus discrimination requiring less cognitive effort, a marker of skill consolidation that SVT may similarly induce over its training block. Importantly, one of these N2 predictions has direct support from within the SVT literature: in elite badminton players, 10 weeks of stroboscopic training shortened motion-onset N2 and N2-r latencies, and these latency changes accounted for more than 60% of the variance in visuomotor reaction time improvement (Hülsdünker et al., 2021). This provides a second directly measured electrophysiological outcome for SVT, alongside the P100 result, and indicates that at least part of the N2 discrimination account is grounded in stroboscopic data rather than inferred.

Alpha band activity provides a complementary electrophysiological perspective on SVT adaptation. Research on sport expertise has shown that athletes exhibit higher occipital peak alpha frequencies at rest than non-athletes, and that this neurophysiological difference mediates superior multiple object tracking performance (Mackenzie et al., 2024). Short-term visual deprivation analogous to the occlusion intervals in SVT has been shown to induce homeostatic shifts in neural gain through monocular deprivation paradigms, with SSVEP measurements confirming that even brief periods of visual occlusion can boost cortical responsiveness to the deprived eye’s input through homeostatic plasticity mechanisms (Lyu et al., 2020). SVT, which creates repeated brief occlusion cycles rather than a single extended deprivation period, may engage similar homeostatic mechanisms in a repeated and potentially cumulative fashion.

**Figure 2:**
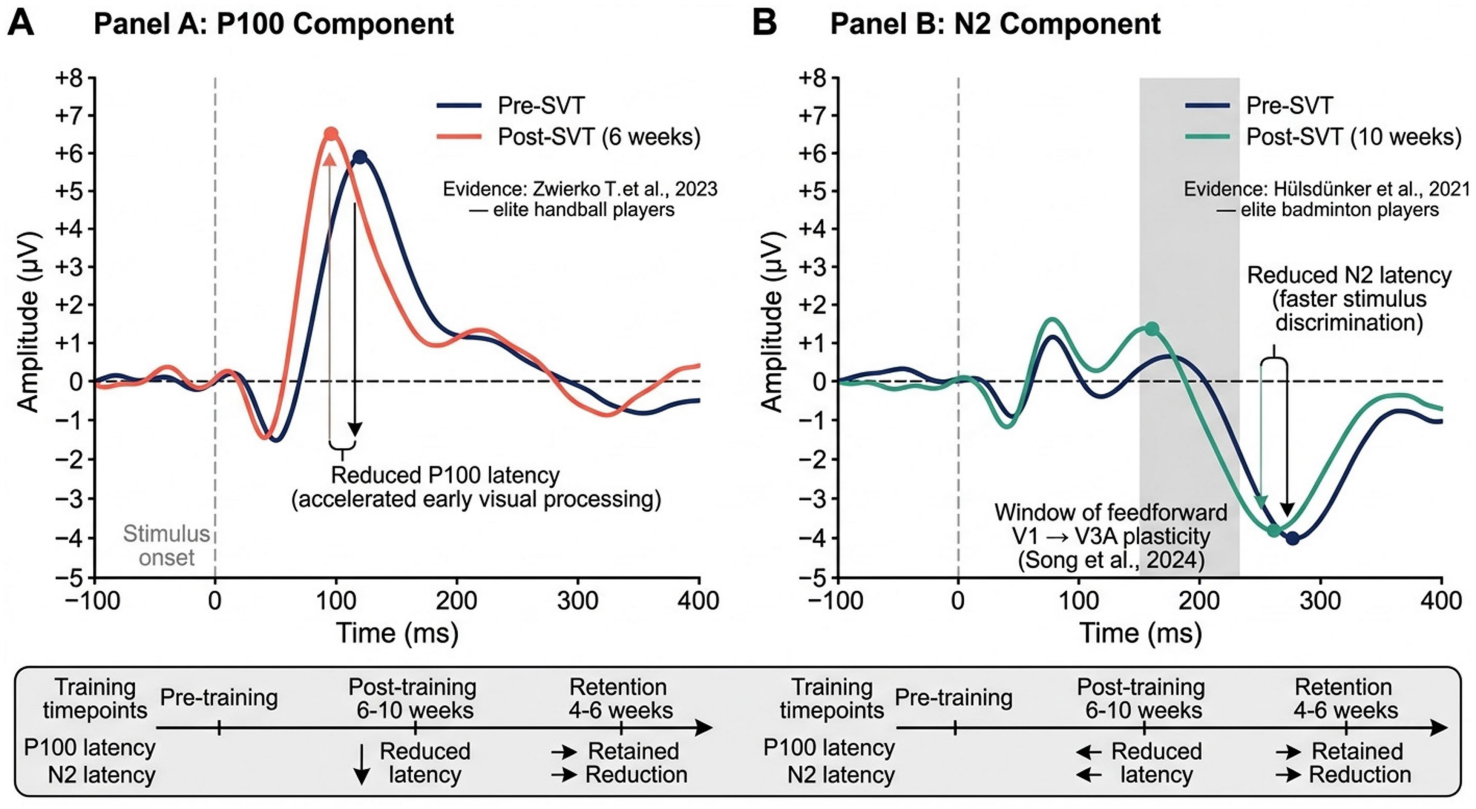
Electrophysiological evidence of stroboscopic visual training: P100 and N2 adaptation timeline.

Sport-specific ERP research identifies additional cortical components likely to be modified by SVT. Expert tennis players show greater evoked potential amplitudes and left medial frontal gyrus activation during processing of sport-specific visual scenes compared to novices, with the frontal difference reflecting enhanced top-down attentional regulation of sport-relevant visual information (He et al., 2018). Table tennis athletes exhibit higher P3 amplitudes and longer latencies than non-athletes during unconscious information processing tasks, indicating greater attentional resource mobilization even outside the sport context (Meng et al., 2025). Research combining locomotion with cognitive tasks shows that environmental visual complexity, including conditions resembling those SVT creates, modulates both P1 and N1 components during concurrent cognitive processing, with attentional resources dynamically reallocated in response to sensory load (Di Bello et al., 2026). Taken together, this evidence suggests SVT may induce a cascade of electrophysiological adaptations spanning from early primary visual cortex processing speed (P100) through intermediate discrimination efficiency (N2) to higher-order attentional allocation (P3), though direct multi-component ERP recording within SVT paradigms remains an open research need. Figure 2 presents a schematic representation of the electrophysiological changes associated with stroboscopic visual training, showing the P100 and N2 components before and after training.

## 5 Perceptual-cognitive adaptations induced by stroboscopic visual training

The behavioral literature on SVT has grown substantially over the past decade, documenting perceptual-cognitive gains across multiple sport populations and a range of outcome domains. Critically, these gains are not uniform: SVT appears to produce specific improvements that depend on the nature of the sport task, the duration of training, and whether SVT is applied acutely or chronically. Understanding this specificity is important for linking the perceptual-cognitive effects of SVT to the underlying neural mechanisms reviewed in the preceding sections.

These effects are also usefully understood through the distinction between near and far transfer, and through attention to the quality of the underlying evidence. Near transfer refers to improvement on tasks that closely resemble the trained task, whereas far transfer refers to generalization to tasks or domains substantially different from those trained. Judged against this distinction, the great majority of demonstrated SVT benefits are near transfer. Gains appear most reliably on outcomes that share the perceptual and motor demands of the stroboscopic drills themselves, such as catching after strobe catching practice (Wilkins and Gray, 2015), reactive agility after agility-based strobe training (Zwierko M. et al., 2024), and postural control after strobe balance training (Lee et al., 2022b). Evidence for genuine far transfer is considerably weaker. Where broader perceptual-cognitive functions have been tested directly, the results are mixed rather than uniformly positive: 8 weeks of stroboscopic training improved anticipation but produced no advantage over control for multiple object tracking or decision-making (Fortes et al., 2025), indicating that benefits did not generalize across perceptual-cognitive domains. The quality of the evidence is also uneven, with most studies using small samples, heterogeneous protocols, and near-transfer outcome measures, and only two supplying direct electrophysiological data. This pattern matters for the neural interpretation offered in this review: training that produces predominantly near transfer is consistent with adaptation in task-specific perceptual and sensorimotor circuits rather than with broad, domain-general change, and claims of wide-ranging cognitive benefit from SVT are not currently supported by the strength or the breadth of the available data.

Reaction speed and visuomotor processing are among the most consistently enhanced outcomes of chronic SVT. Zwierko M. et al. (2023) conducted a six-week randomized controlled trial with 50 youth volleyball players and found that the SVT group improved significantly more than controls on complex reaction speed (Cohen’s d = 0.87) and simple motor time (d = 0.42) at post-test, with partial retention at 4 weeks. A subsequent SVT study with the same volleyball population showed that stroboscopic training also enhanced visuomotor reaction speed in goal-directed agility movements, with saccade acceleration changes significantly correlated with visuomotor reaction gains (Zwierko T. et al., 2023). This correlation implicates improvements in oculomotor control as a potential neural mediator of the visuomotor benefits of SVT, linking the training effect to changes in the visual-motor pathway rather than only to higher cognitive processes.

Anticipatory skill is a domain where SVT appears to exert particularly selective effects. Fortes (Fortes et al., 2025) studied 28 male soccer players over 8 weeks of SVT and found that the stroboscopic group showed superior anticipation skill at post-test compared to sport-specific practice controls, even though multiple object tracking and decision-making scores improved in both groups without a between-group difference. This dissociation between anticipation and decision-making effects suggests that SVT acts specifically on the perceptual stage of information processing, namely the generation of accurate forward predictions about opponent and ball movement, rather than on the cognitive stage of response selection from among identified options (Ballester et al., 2017; Fortes et al., 2025). This interpretation aligns directly with the predictive coding account of SVT: repeated occlusion trains the brain to generate better anticipatory representations of ongoing motion, which manifests behaviorally as more accurate anticipation without necessarily accelerating deliberate decision processes.

Visual attention and motion perception are further targets of SVT. Wilkins and Gray (2015) found that regardless of group assignment, athletes whose useful field of view and motion-in-depth sensitivity scores improved after stroboscopic catching drills also improved most in catching performance, demonstrating a mechanistic link between specific perceptual changes and sport skill. Reactive agility, which requires both rapid perceptual detection of a movement cue and quick directional response, has been consistently improved by chronic SVT in both volleyball (Zwierko M. et al., 2024) and soccer contexts (Fortes et al., 2025). The acutely applied form of SVT during warm-up shows more context-specific effects: in soccer players, stroboscopic warm-up significantly enhanced reactive agility for ball dribbling tasks (effect size = 0.57) but produced no significant improvement for agility without ball contact, illustrating that the match between stroboscopic training conditions and the subsequent task context determines whether acute SVT benefits transfer (Zwierko T. et al., 2023). In contrast, stroboscopic warm-up in elite table tennis athletes failed to improve reaction time beyond the warm-up effect achieved under normal visual conditions (Strainchamps et al., 2023), reinforcing the finding that short-term SVT effects are task-specific and that chronic training is likely necessary for robust neural adaptation.

SVT also produces consistent improvements in balance and sensorimotor control when embedded in balance training protocols. Balance training augmented with stroboscopic glasses in chronic ankle instability patients produced significantly greater improvements in postural control compared to standard balance training alone, with the stroboscopic group showing altered visual reliance patterns indicating that the central nervous system had recalibrated its weighting of sensory inputs (Lee et al., 2022a; Mohess et al., 2024). These postural improvements reflect the sensory reweighting mechanism described in Section 2: SVT during balance tasks forces greater reliance on proprioceptive and vestibular signals (Kim et al., 2017), which over repeated training sessions may strengthen the sensorimotor integration circuits that handle balance control when visual information is degraded or absent, a scenario commonly encountered in sport (Grooms et al., 2018; Harrison et al., 2024).

A comprehensive synthesis of 14 SVT studies involving 542 athletes concluded that SVT consistently produces improvements in reaction time, hand-eye coordination, agility, and reactive agility, with moderate and consistent sport-specific performance gains across the literature (Dingirdan Gultekinler et al., 2025). The strongest and most consistent evidence is for reaction time and visuomotor performance, precisely the domains where the P100 acceleration and N2 efficiency improvements reviewed electrophysiologically would be expected to manifest behaviorally. Balance and jump performance improvements were documented in several studies, extending the reach of SVT effects beyond the purely perceptual into the sensorimotor domain. The breadth of these effects across reaction speed, anticipation, visuomotor control, and balance underscores that SVT does not act on a single perceptual mechanism but engages a distributed network of circuits involved in visual prediction, sensory integration, and motor preparation. Table 2 summarizes the design features, populations, training protocols, and neurophysiological outcomes of all published SVT intervention studies. Although the balance of published evidence favors positive effects of stroboscopic visual training, a comprehensive appraisal must also weigh the studies reporting null, inconsistent, or unfavorable results, both to guard against confirmation bias and to define the boundary conditions of the intervention. The clearest null findings come from acute, warm-up applications. In highly trained table tennis athletes, a stroboscopic warm-up produced no improvement in sport-specific reaction speed beyond an equivalent warm-up under normal vision (Strainchamps et al., 2023). When stroboscopic eyewear was applied acutely before a soccer agility task, reactive agility improved only for the ball-dribbling condition and showed no benefit when the task did not involve ball contact (T. Zwierko et al., 2024). Effects within chronic-training studies are also frequently selective rather than global: 8 weeks of stroboscopic training in soccer players improved anticipation but produced no between-group advantage for multiple object tracking or decision-making (Fortes et al., 2025), indicating that the training does not transfer uniformly across perceptual-cognitive domains. Protocol comparisons have been similarly inconclusive, with progressively increasing and constant occlusion schedules yielding no reliable difference in catching gains (Wilkins and Gray, 2015), so the field cannot yet specify which parameters are responsible for adaptation.

Some results indicate not merely an absence of benefit but that stroboscopic conditions can reduce performance, or that gains depend on the specific occlusion method. In a laboratory comparison of techniques, intermittent stroboscopic vision maintained multiple object tracking performance whereas full goggle occlusion impaired it, showing that perturbing vision rather than eliminating it is what preserves function and that not all visual interruption is beneficial (Bennett et al., 2018). In female collegiate volleyball players, a slower strobe frequency during depth jumps reduced reactive strength index and jump height relative to normal vision, indicating that stroboscopic disruption can acutely lower movement output (Kroll et al., 2023). Where retention has been assessed, gains have only partially persisted and had decayed by 4 weeks after training (Zwierko M. et al., 2023; Zwierko T. et al., 2023), so durability should not be assumed. These observations do not overturn the generally favorable behavioral evidence, but they qualify it: benefits are most reliable for chronic rather than acute protocols, are domain-specific rather than uniform, and depend on the trained condition matching the target task. The small size of the literature, together with the greater ease of publishing positive than null results, further suggests that the true distribution of effects may be less uniformly positive than the published record implies. We therefore regard stroboscopic visual training as a reliable but bounded and context-dependent intervention rather than a universally effective one.

## 6 Neuroplasticity mechanisms: how stroboscopic visual training may reshape the brain

The perceptual-cognitive and electrophysiological changes produced by SVT must ultimately be explained by modifications in neural circuitry. Although no published study has directly measured cortical structural or functional changes following SVT using fMRI or high-density EEG source localization, multiple converging lines of evidence from perceptual learning neuroscience and sport neuroscience suggest plausible plasticity mechanisms that SVT is well positioned to engage. Table 1 maps each proposed plasticity mechanism against its supporting evidence base, the specific stroboscopic visual training condition that engages it, and whether the evidence comes directly from a stroboscopic visual training study or is inferred from the broader perceptual learning and sport neuroscience literature. The mechanisms proposed in this section follow a common inferential structure that we make explicit here rather than restating at each step. In each case, a form of plasticity has been demonstrated in a related paradigm, most often visual perceptual learning, sensory deprivation, or motor learning, and we argue that the specific demands SVT imposes are of the kind known to engage that mechanism. This makes each mechanism a testable prediction about SVT, not a description of an effect already observed under stroboscopic conditions. The strength of each prediction depends on how closely the training demands of the source paradigm match those of SVT, and we flag this correspondence, and its limits, where it is weakest. None of the mechanisms below has been confirmed in an SVT study, and all are classified as plausible rather than demonstrated in Table 1.

Visual perceptual learning, the sustained improvement in visual task performance driven by training or experience, is now established as a phenomenon involving genuine cortical reorganization even in the adult brain (Song et al., 2024). Song et al. (2024) used MEG to show that training on visual motion direction discrimination increased decoding accuracy from early visual cortex signals, reduced decoding latency, and strengthened feedforward connectivity from primary visual cortex to V3A within the 160 to 230 ms window after stimulus onset. This temporal profile maps closely onto the P100 latency window in which SVT has been shown to produce improvements (Zwierko T. et al., 2023), suggesting that SVT may drive similar feedforward plasticity in early and extrastriata visual cortex through its repeated motion encoding demands. Chen et al. (2016) provided complementary evidence that perceptual training on coherent motion causes a functional shift from MT toward V3A as the dominant cortical site for motion decoding, demonstrating that training can reorganize the relative weighting of visual areas for a trained stimulus. Given that SVT involves repeated encoding of interrupted motion trajectories, analogous reorganization of motion-processing areas is a plausible outcome.

Short-term visual deprivation provides a mechanistic parallel that is particularly relevant to the occlusion intervals in SVT. Lyu et al. (2020) demonstrated using SSVEP that even 2 hours of monocular visual deprivation boosts neural gain for the deprived eye through homeostatic plasticity, with the SSVEP amplitude ratio for the deprived eye increasing progressively across the deprivation period. SVT creates repeated brief occlusion intervals rather than extended monocular deprivation, but the underlying principle of homeostatic upregulation of cortical responsiveness following visual input reduction may operate across both timescales. At the level of cortical laminar circuits, Jia et al. (2020) showed using 7T fMRI that perceptual learning alters orientation-selective representations specifically in the superficial layers of V1, consistent with plasticity mediated by horizontal recurrent connections. SVT, through its repeated cycles of sensory interruption and reacquisition, may similarly strengthen recurrent connections between motion-selective neurons in MT/V5 and predictive parietal inputs, reshaping the laminar structure of processing in these areas (Jia et al., 2020; Ni et al., 2023).

The cerebellum is a further site of likely SVT-induced plasticity, particularly for the timing precision required in interceptive tasks. Chen et al. (2020) showed that the right anterior cerebellum was differentially activated for correct versus incorrect anticipatory decisions in baseball batters across all expertise levels, supporting its role in fine-tuning forward models for interceptive timing. Chen et al. (2023) extended these findings to show that perceptual experience in batters, rather than motor experience in pitchers, specifically activated the ventral extrastriata cortex during pitch trajectory prediction, linking visual expertise to distinct neural substrates from motor expertise. Muraskin et al. (2015) found that expert batters showed enhanced contingent negative variation and fusiform gyrus activation during go/no-go decisions, indicating that perceptual-action coupling circuits are specifically shaped by interceptive batting experience. SVT, by imposing repeated prediction demands across a long series of occlusion cycles embedded in sport-specific practice, provides a systematic training signal for these predictive circuits that may be more concentrated and cognitively demanding than standard practice alone.

Cross et al. (2013) demonstrated that visual training on occluded action sequences increased inferior parietal, superior temporal, and cerebellar activation during prediction of trained stimuli, indicating that training redistributes cortical resources toward prediction-relevant regions. This redistribution is consistent with SVT’s mechanism: by systematically requiring the brain to predict rather than perceive, SVT may shift the balance of processing resources toward predictive parietal and cerebellar networks and away from passive early visual encoding regions. The sensorimotor plasticity research from SVT balance studies reinforces this interpretation. Stroboscopic visual training during landing tasks alters ground reaction force profiles and lower-extremity muscle activation patterns, indicating that the brain’s sensorimotor integration circuitry may reconfigure its feedforward motor commands when trained under conditions of visual uncertainty (Grooms et al., 2018; Harrison et al., 2024). Taken together, these findings are consistent with a proposed multi-level model of SVT-related neuroplasticity spanning early visual cortex feedforward speed, extrastriata area functional reorganization, recurrent laminar plasticity, cerebellar forward model refinement, and sensorimotor integration reweighting.

## 7 Training parameters and their neural implications for stroboscopic visual training

One of the most practically relevant yet insufficiently resolved questions in the stroboscopic visual training literature concerns how specific training parameters, namely session duration, occlusion frequency, total volume, and integration with sport-specific practice, determine the magnitude and direction of neural adaptation. Across published studies, stroboscopic visual training protocols differ substantially in these parameters, making it difficult to derive firm dose–response relationships or to compare neural outcomes across trials (Ballester et al., 2017; Dingirdan Gultekinler et al., 2025). It should be stated directly that no study has systematically varied a single training parameter while holding the others constant, which is the design required to establish a dose–response relationship. The parameter effects discussed below are therefore inferred from comparisons across heterogeneous protocols rather than from controlled dose–response data, and they should be read as provisional.

Session duration and total training weeks are among the most consistently reported parameters in stroboscopic visual training research. The six-week block is the most frequently used design. Zwierko T. et al. (2023) showed that 6 weeks of stroboscopic visual training in elite handball players produced significant reductions in P100 implicit time, with the electrophysiological improvements partially retained at a four-week follow-up, though the gains were smaller at retention than immediately after training. Zwierko T. et al. (2023) applied an identical six-week stroboscopic visual training protocol to volleyball players and found that complex reaction speed effects (d = 0.87) were larger immediately post-training than at the four-week retention point. Fortes et al. (2025) extended the stroboscopic visual training period to 8 weeks with soccer players and found that superior anticipatory skill in the stroboscopic group was maintained at post-test, though the absence of a longer follow-up limits conclusions about persistence. Across these studies, the convergent picture is that 6 to 8 weeks of stroboscopic visual training produces measurable perceptual-cognitive change, but some gains decay without continued stroboscopic exposure, pointing to the likely necessity of maintenance phases in applied programmed (Zwierko M. et al., 2023; Zwierko T. et al., 2023).

The occlusion frequency setting, which controls how completely and how often visual feedback is removed during stroboscopic visual training, has direct implications for the depth of cortical engagement. Wilkins and Gray (2015) compared a variable strobe rate stroboscopic visual training protocol, in which occlusion duration was progressively increased across nine catching sessions, against a constant strobe rate protocol. Neither produced significantly greater performance gains than the other, suggesting that the overall level of visual demand rather than the specific progression structure may be the more influential variable. From a cortical standpoint, this matters because the depth of perceptual learning-induced plasticity is known to scale with task difficulty: more demanding discrimination tasks engage broader and higher-level visual areas and produce more generalizable cortical reorganization (Jia et al., 2020; Ni et al., 2023). Applying this principle to stroboscopic visual training, progressively increasing the occlusion frequency across sessions may impose escalating demands on motion extrapolation and predictive coding circuits in MT/V5 and V3A, potentially driving deeper cortical adaptation than a static difficulty level (Ni et al., 2023; Song et al., 2024).

Whether stroboscopic visual training is administered in isolation or embedded within sport-specific practice is another parameter with neural implications. Standard stroboscopic visual training protocols embed the stroboscopic eyewear within sport-specific motor tasks, ensuring that the predictive circuits trained are engaged in the same attentional and motor context in which they will later be required to operate. The thalamo-sensorimotor functional connectivity that underlies elite motor performance is known to strengthen specifically through years of domain-relevant training (Huang et al., 2017), suggesting that stroboscopic visual training contextualized within sport-specific movement may leverage these established circuits more effectively than isolated laboratory-based stroboscopic practice. Anguera et al. (2025) demonstrated this principle in a different modality: a virtual reality cognitive training programmed supplementing regular tennis practice produced significantly larger performance rating improvements than sport practice alone, reinforcing the principle that perceptual-cognitive interventions produce additive benefits when embedded within relevant motor experience. Woost et al. (2018) found no additive transfer when physical and spatial training were administered sequentially rather than in an integrated format, suggesting that the contextual alignment of stroboscopic visual training with sport-specific motor tasks is not merely convenient but neurobiologically necessary for optimal transfer (Fortes et al., 2025).

A final consideration is more speculative and is offered explicitly as a direction for future work rather than as an established parameter of SVT. Sleep-dependent memory consolidation has not been measured in any stroboscopic visual training study, and no direct evidence links it to SVT outcomes. We raise it because SVT programs necessarily span multiple weeks and therefore include repeated sleep intervals, and because sleep is known to support the offline consolidation of motor and perceptual learning in other contexts. Motor and perceptual skill improvements acquired during practice are known to consolidate offline, with sleep selectively benefiting goal-directed procedural learning (Pace-Schott and Spencer, 2013). Disrupted sleep prevents the overnight performance gains characteristic of healthy sleepers following motor skill practice (Cellini et al., 2014). Stroboscopic visual training protocols that span multiple weeks necessarily involve repeated sleep intervals between sessions, and the cortical changes indexed by P100 latency reductions and N2 modifications may accumulate partly through sleep-dependent processes. The broader literature on exercise and cognitive function also suggests that training parameters including intensity and frequency must be calibrated to allow adequate recovery for neuroplastic consolidation to occur (Chow et al., 2021). Future stroboscopic visual training research should therefore incorporate sleep quality assessment as a standard covariate to control for its likely influence on between-session neural consolidation.

## 8 Limitations of the stroboscopic visual training literature and future directions

Despite the accumulating behavioral evidence supporting stroboscopic visual training as a practical perceptual-cognitive intervention, the current literature has several important limitations that prevent strong mechanistic conclusions. These limitations are not trivial gaps in an otherwise mature body of evidence they represent foundational absences that currently prevent stroboscopic visual training from being fully characterized as a neuroscience-validated training tool.

The most critical limitation is the near-total absence of direct neuroimaging data from stroboscopic visual training experiments. The visual evoked potential study of elite handball players by Zwierko T. et al. (2023) is one of only two published investigations linking stroboscopic visual training to a direct electrophysiological brain measure in a controlled design, in this case reduced P100 implicit time; the other is a badminton study reporting motion-onset N2 latency changes and premotor-time shortening (Hülsdünker et al., 2021). Aside from these two datasets, every other study in the stroboscopic visual training literature has characterized training effects purely through behavioral outcomes such as reaction time, catching success, and agility scores.

Sample sizes in stroboscopic visual training research are consistently small. Most published trials include between 20 and 50 participants, limiting statistical power and precluding meaningful subgroup analyses. Zwierko M. et al. (2023) noted gender-related differences in the stroboscopic visual training response, with female volleyball players showing stronger gains in saccade dynamics and reactive agility, but acknowledged that the sample was insufficient to draw firm conclusions. Age, baseline visual processing efficiency, and sport expertise level are all plausible moderators of the neural response to stroboscopic visual training, yet none has been systematically examined. Without adequately powered stratified designs, the optimal stroboscopic visual training dose for different populations cannot be identified.

Longitudinal follow-up beyond 4 weeks is absent from all stroboscopic visual training studies. The retention assessments conducted by Zwierko T. et al. (2023) are the longest available, and both showed partial decay of stroboscopic visual training-induced gains. Whether the cortical changes proposed to underlie stroboscopic visual training adaptations persist, consolidate further, or revert entirely after training cessation is entirely unknown. Athletes require durable adaptations, not transient performance boosts, and the durability of stroboscopic visual training-induced neural change remains completely unaddressed in the published literature.

Protocol standardization across stroboscopic visual training studies is poor. Studies differ in the strobe device used, its frequency settings, the sport-specific tasks employed, the total number of sessions, and the outcome measures collected. This variability makes direct comparisons across studies difficult and quantitative synthesis unreliable. Establishing agreed minimum reporting standards for stroboscopic visual training research, specifying device settings, session structure, embedding in sport practice or isolation, and neurophysiological measures where available, would substantially improve the cumulative value of future work.

Future stroboscopic visual training research priorities should therefore include the following. First, randomized controlled trials combining stroboscopic visual training with concurrent EEG recording and longitudinal follow-up of at least 6 months would allow electrophysiological markers of neural adaptation to be tracked alongside behavioral outcomes and their durability to be assessed. Second, fMRI studies measuring pre-post stroboscopic visual training responses in MT/V5, V3A, posterior parietal cortex, and cerebellum would directly test the plasticity mechanisms proposed in this review. Third, stratified designs examining stroboscopic visual training response as a function of expertise, age, and sex would clarify which athlete populations benefit most and whether training parameters need to be individually adjusted. Fourth, systematic dose–response studies varying occlusion frequency and session volume would provide normative data for evidence-based stroboscopic visual training prescription. Finally, sleep quality should be monitored throughout stroboscopic visual training programs to account for its likely influence on between-session neural consolidation.

## 9 Conclusion

This narrative review has synthesized current evidence on the neural and electrophysiological mechanisms that may underlie the effects of stroboscopic visual training in interceptive sports. Stroboscopic visual training operates by periodically removing visual feedback during sport-specific motor tasks, thereby compelling the central nervous system to sustain internal predictions of moving targets rather than passively receiving real-time sensory information. This mechanism aligns directly with predictive coding frameworks and places stroboscopic visual training within a coherent neuroscientific account of how visual processing can be trained through demand rather than enrichment.

The electrophysiological evidence most directly relevant to stroboscopic visual training, particularly the P100 implicit time reductions reported following stroboscopic visual training in handball players (Zwierko T. et al., 2023), is consistent with acceleration of early visual pathway transmission that is also observed in perceptual learning studies using MEG and fMRI. The N2 and P3 modifications documented in related perceptual and motor training contexts suggest that stroboscopic visual training may additionally refine later cortical discrimination stages, though this has not yet been directly measured within a stroboscopic visual training paradigm. The perceptual-cognitive gains observed across stroboscopic visual training studies, including faster complex reaction times, improved anticipatory skill, and enhanced visuomotor performance, reflect the combined influence of these early and later cortical adaptations and are task-specific in their expression.

The most important conclusion of this review is also the most sobering: the neuroscience of stroboscopic visual training is largely a field of plausible inference rather than demonstrated mechanism. The behavioral evidence for stroboscopic visual training is real and replicable, but the cortical story rests almost entirely on extrapolation from adjacent literatures. Confirming that stroboscopic visual training produces the neural changes it is theorized to produce requires EEG and fMRI studies that have not yet been conducted. Until they are, stroboscopic visual training should be regarded as a promising evidence-informed tool whose neuroscientific foundations remain an active and open research question.
